# Supplementary material for: Psychometric Testing and Validation of the Italian Version of the Helsinki Chronic Pain Index (I-HCPI) in Dogs with Pain Related to Osteoarthritis
Source: Animals (Basel). 2023 Dec 25;14(1):83. doi: 10.3390/ani14010083 (PMC10778034; doi:10.3390/ani14010083)
Supplement: Supplementary file 1 [file animals-14-00083-s001.zip › animals-2760432-supplementary.pdf]

CeRiDA
Centro di Ricerca sul Dolore Animale

HELSINKI CHRONIC PAIN INDEX, ITALIAN VERSION (I-HCPI)

QUESTIONARIO PER PROPRIETARI

Data ________________________________ Questionario no. ________________________

Nome del cane ________________________ Diagnosi ______________________________

Proprietario ___________________________ Firma ________________________________

Seleziona solo una risposta, quella che meglio descrive il tuo cane nella settimana scorsa

1) In termini di umore, il tuo cane è:

Molto vivace	vivace	né vivace né indifferente	indifferente	molto indifferente
☐	☐	☐	☐	☐

2) Il tuo cane gioca:

Molto volentieri	volentieri	riluttante	molto riluttante	non gioca per niente
☐	☐	☐	☐	☐

3) Il tuo cane vocalizza (uggiola, si lamenta o piange):

mai	quasi mai	a volte	spesso	molto spesso
☐	☐	☐	☐	☐

4) Il tuo cane cammina:

Con grande facilità	con facilità	né con facilità né con difficoltà	con difficoltà	con grande difficoltà
☐	☐	☐	☐	☐

5) Il tuo cane trotta:

Con grande facilità	con facilità	né con facilità né con difficoltà	con difficoltà	con grande difficoltà
☐	☐	☐	☐	☐

6) Il tuo cane galoppa:

Con grande facilità	con facilità	né con facilità né con difficoltà	con difficoltà	con grande difficoltà
☐	☐	☐	☐	☐

CeRiDA
Centro di Ricerca sul Dolore Animale

7) Il tuo cane salta (ad es. in macchina, sul divano…):

Con grande facilità	con facilità	né con facilità né con difficoltà	con difficoltà	con grande difficoltà
☐	☐	☐	☐	☐

8) Il tuo cane si corica:

Con grande facilità	con facilità	né con facilità né con difficoltà	con difficoltà	con grande difficoltà
☐	☐	☐	☐	☐

9) Il tuo cane si alza da steso:

Con grande facilità	con facilità	né con facilità né con difficoltà	con difficoltà	con grande difficoltà
☐	☐	☐	☐	☐

10) Il tuo cane si muove dopo essere stato fermo a lungo:

Con grande facilità	con facilità	né con facilità né con difficoltà	con difficoltà	con grande difficoltà
☐	☐	☐	☐	☐

11) Il tuo cane si muove dopo un’attività molto intensa o esercizi più pesanti:

Con grande facilità	con facilità	né con facilità né con difficoltà	con difficoltà	con grande difficoltà
☐	☐	☐	☐	☐

Grazie per la tua collaborazione

Note del veterinario:

____

____

____

____

____
